# Prevalence of Pancreaticobiliary Maljunction and High Confluence of the Pancreaticobiliary Ducts: True Incidence of Gallbladder Cancer

**DOI:** 10.1002/jhbp.12187

**Published:** 2025-07-07

**Authors:** Shintaro Shirai, Kenitiro Kaneko, Shoko Kato, Remi Kondo, Takaaki Osawa, Yasuyuki Fukami, Tsuyoshi Sano

**Affiliations:** ^1^ Department of Gastroenterological Surgery Aichi Medical University Nagakute Japan

**Keywords:** biliary tract neoplasm, incidence, magnetic resonance cholangiopancreatography, pancreaticobiliary maljunction, prevalence

## Abstract

**Background:**

Although pancreaticobiliary maljunction (PBM) is associated with a high incidence of biliary cancer, it often goes undiagnosed. This means that the true prevalence of PBM and the incidence of biliary cancer are unknown. High confluence of the pancreaticobiliary ducts (HCPBD) may be an intermediate PBM variant, though reports are scarce. In this study, we aimed to determine the true prevalence of PBM and HCPBD and the actual incidence of biliary cancer.

**Methods:**

We retrospectively analyzed data from adults who underwent cholecystectomy for benign gallbladder disease and compared them to those with gallbladder and bile duct cancers. The common channel (CC) and narrow distal segment (NDS) were measured using magnetic resonance cholangiopancreatography to diagnose PBM and HCPBD.

**Results:**

PBM and HCPBD were identified in 0.44% and 0.88% of 2046 benign cholecystectomies, 16% and 4.2% of gallbladder cancers, and 1.3% and 3.8% of bile duct cancers, respectively (*p* < 0.01).

**Conclusions:**

The overall prevalence was 0.44%. Combined with national data, the gallbladder cancer incidence in PBM was estimated to be 2.4% over one decade, which is 38‐fold higher than that in the general population. Approximately 1% of the population have HCPBD, which may be a risk factor for biliary cancer.

## Graphical abstract

High gallbladder cancer incidence is linked to nondilated pancreaticobiliary maljunction, yet asymptomatic cases remain underrecognized. In over 2000 cases of cholecystectomy for benign disease, Shirai and colleagues applied preoperative magnetic resonance cholangiopancreatography to identify pancreaticobiliary maljunction and high pancreaticobiliary confluence, enabling more accurate estimation of the true gallbladder cancer incidence.

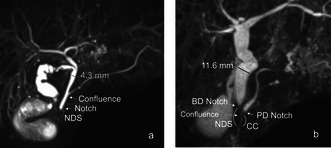

## 1 Introduction

Pancreaticobiliary maljunction (PBM), also known as anomalous pancreaticobiliary union, is a malformation in which the pancreaticobiliary ducts converge outside the duodenal wall and thus the sphincter of Oddi, resulting in mutual regurgitation of bile and pancreatic juice, which impacts the pancreaticobiliary system [1]. Biliary carcinogenesis is frequently associated with PBM, with the incidence of biliary cancer reaching 42% in adults with PBM without bile duct dilatation (nondilated PBM) [2]. However, this incidence may be overreported because many patients with nondilated PBM are asymptomatic and remain undiagnosed. Adults with nondilated PBM are typically asymptomatic unless their cancer is advanced, or they have symptomatic gallstones [3]. Additionally, inexperienced physicians are often unfamiliar with PBM, even in Japan, where the prevalence of PBM is higher than that in other regions, resulting in more undiagnosed cases.

An intermediate anomaly between the PBM and normal pancreaticobiliary junction and high confluence of the pancreaticobiliary ducts (HCPBD) has recently been recognized. HCPBD is defined by a common channel (CC) length of ≥ 6 mm with the pancreaticobiliary junction still positioned within the contractile area of the sphincter of Oddi [4]. HCPBD permits pancreatic juice reflux into the biliary tract, though to a lesser extent than PBM. Kamisawa et al. [5] reported that HCPBD can also cause gallbladder cancer and precancerous epithelial changes, as observed in PBM; however, beyond that report, the available literature on HCPBD remains limited, and the prevalence and pathophysiology of HCPBD remain unclear [6].

Therefore, this study aimed to determine the actual incidence of carcinogenesis in patients with PBM and the true prevalence and pathophysiology of HCPBD through a retrospective review of patients who underwent cholecystectomy for benign gallbladder disease. We then compared these data with those from patients who underwent surgery for gallbladder and/or bile duct cancer.

## 2 Methods

### 2.1 Subjects

A total of consecutive 2068 adult patients (age range, 19–98 years) underwent cholecystectomy for benign gallbladder disease between January 2013 and March 2024 in our department, of whom 11 underwent non‐imaging evaluations and six had inconclusive imaging results, while pathological examinations revealed incidental gallbladder cancer in five patients. After excluding these 22 patients, the medical records of the remaining 2046 patients were retrospectively reviewed (Table 1). Cholecystectomy was performed for gallstones and/or cholecystitis in 1998 patients, polyps or adenomyomatosis in 44, and torsion of the gallbladder in four. In the same 11‐year period, 49 patients with gallbladder cancer (including the five aforementioned incidental cancers) and 79 with bile duct cancer were treated in our department. Their medical records were also analyzed for comparison. In patients with PBM and HCPBD with benign gallbladder diseases, microscopic slides of the gallbladder prepared after surgery were reexamined, although sections were not from the entire gallbladder unless the pathologist had judged it necessary. The prognoses of patients with PBM and HCPBD who underwent cholecystectomy for benign gallbladder disease were obtained from the medical records or via telephone. This study was approved by our institutional review board (approval no: 2024–183). Informed consent was waived due to the retrospective nature of this study.

**TABLE 1:** Frequency of pancreaticobiliary maljunction and high confluence of the pancreaticobiliary ducts.

	Benign GB disease	GB cancer	BD cancer	Total
	*n* = 2046	*n* = 49	*n* = 79	*n* = 2174
Age (years), median (range)	62 (19–98)	71 (53–90)	72 (34–88)	
M:F	1113:933 (1.2:1)	26:23	52:27	
Pancreaticobiliary maljunction, *n* (%)	9 (0.44%)^a^	8 (16%)^a^, ^b^	1 (1.3%)^b^	18
Age (years), median (range)	50 (19–71)	67 (54–79)	79	58 (19–79)
M:F	2:7	1:7	0:1	3:15
Preoperative diagnosis, *n* (%)	5 (56%)	6 (75%)	1 (100%)	12 (67%)^c^
High confluence of pancreaticobiliary ducts, *n* (%)	18 (0.88%)	2 (4.2%)	3 (3.8%)	23
Age (years), median (range)	60 (31–83)	72, 85	75, 79, 80	63 (31–85)
M:F	2:16	1:1	3:0	6:17
Preoperative diagnosis, *n* (%)	4 (22%)	0	0	4 (17%)^c^

Abbreviations: BD, bile duct; F, female; GB, gallbladder; M, male.

^a^ *p* < 0.01.

^b^ *p* < 0.05 using Fisher's exact test with Bonferroni adjustment.

^c^ *p* = 0.0031 using Fisher's exact test.

### 2.2 Diagnostic Imaging Assessments and Definitions

**FIGURE 1:**
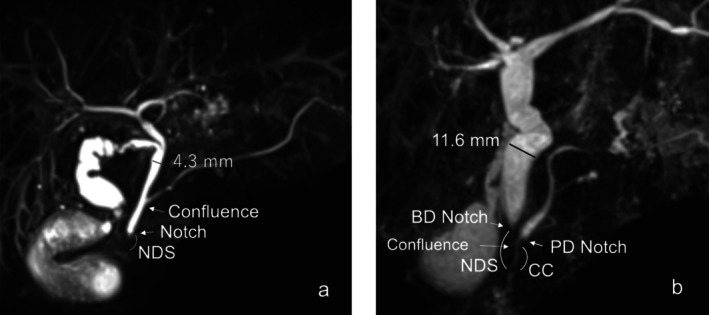
Representative cases of pancreaticobiliary maljunction (PBM) (a) and high confluence of the pancreaticobiliary duct (HCPBD) (b). (a) Notch indicates the area where the sphincter of Oddi contracts (narrow distal segment, NDS) and is clearly visible within the common channel (CC). (b) Notch in the bile duct (BD) indicates the NDS where the sphincter of Oddi is located. A notch was also identified in the pancreatic duct (PD) of this patient, which is typically obscure in most patients. Confluences between BD and PD were identified in the NDS. The CC length were 8.5 mm and NDS length was 14.5 mm.

Magnetic resonance cholangiopancreatography (MRCP) examinations were performed using 1.5T systems (MAGNETOM Aera, Siemens, Germany) or 3T systems (MAGNETOM Skyla, Siemens) with a three‐dimensional thin multislice prospective acquisition correction technique. The lengths of the CC and narrow distal segment (NDS) created by the sphincter of Oddi were measured using MRCP and, if available, direct visualization using endoscopic retrograde cholangiopancreatography (ERCP) and/or intraoperative cholangiography. Patients were diagnosed with PBM when the length of the CC was ≥ 15 mm or when a notch existed within the CC (CC >NDS) (Figure 1) [7, 8, 9], whereas they were diagnosed with HCPBD when the length of the CC was ≥ 6 mm with the pancreaticobiliary ductal confluence still within the NDS (CC <NDS) (Figure 1). The relevant images were screened by two authors (SS and SK) and judged by a third author (KK, with more than 30 years of experience in this field). Among patients diagnosed with PBM or HCPBD, the widest length of the extrahepatic bile duct was measured using MRCP, except in those with bile duct cancer, among whom these analyses were only possible using ERCP images. Bile duct dilatation was determined based on the sonographic upper limit values of the extrahepatic bile duct diameter, which varied depending on the patient's age [10].

### 2.3 Statistical Analysis

Chi‐squared and Fisher's exact tests were used for statistical analysis, with statistical significance set at *p* < 0.05. For repeated comparisons, the significance level was adjusted using the Bonferroni method.

## 3 Results

The frequencies of PBM and HCPBD in each group are presented in Table 1. Both PBM and HCPBD were significantly more frequent in patients with biliary cancer (nine [7.0%] and five [3.9%] of 128 patients, respectively) than in those with benign gallbladder disease (nine [0.44%] and 18 [0.88%] of 2046 patients, respectively) (*p* < 0.01), whereas the frequency of PBM was highest among patients with gallbladder cancer (eight [16%] of 49 patients). Among the 18 patients with PBM, eight (44%) were diagnosed with gallbladder cancer, while only one (5.6%) was diagnosed with bile duct cancer. Among the 23 patients with HCPBD, two (8.7%) were diagnosed with gallbladder cancer and three (13%) with bile duct cancer. PBM and HCPBD predominantly affected women (1:5 and 1:2.8 men: women, respectively) and both were frequently undiagnosed before surgery, although HCPBD was diagnosed significantly less frequently than PBM (*p* = 0.0031).

The CC and NDS lengths and bile duct diameters of the patients diagnosed with PBM and HCPBD are listed in Table 2. The bile duct was normal or slightly dilated in 38 (93%) of the 41 patients with PBM or HCPBD. Bile amylase levels were measured in four patients with PBM in the benign cholecystectomy group, with values of 69 871, 23 365, 439, and 28 IU/L, and in four patients with PBM in the gallbladder cancer group, with values of 13 118, 18 260, 90 473, and 168 856 IU/L. For the three patients with HCPBD in the benign cholecystectomy group, the amylase levels were 24 222, 320, and 26 IU/L.

**TABLE 2:** Common channel and narrow distal segment lengths and bile duct diameters in patients with PBM and HCPBD.

Groups	*n*	Common channel length, median (range) (mm)	Narrow distal segment length, median (range) (mm)	Gallstones, *n* (%)	BD diameter			
					Within UL	0–2 mm above UL	2–4 mm above UL	CBD
PBM in benign GB diseases	9	11.2 (10.1–22.1)	7.8 (5.6–10.8)	7 (78%)	6	2	1	
PBM in GB cancers	8	15.7 (12.5–22.8)	9.9 (8.1–10.6)	0	2	3	1	2
PBM in BD cancers	1	21.7	12.2	0				1
HCPBD in benign GB diseases	18	7.8 (6.0–12.0)	11.9 (9.2–15.2)	15 (79%)	10	5	3	
HCPBD in GB cancers	2	6.7, 7.4	11.0, 13.9, respectively	1 (50%)		2		
HCPBD in BD cancers	3	7.4, 8.5, 9.3	12.1, 11.5, 11.8, respectively	1 (33%)	1^a^	1^a^	1^a^	
Total	41				19	13	6	3

Abbreviations: BD, bile duct; CBD, congenital biliary dilatation; GB, gallbladder; HCPBD, high confluence of the pancreaticobiliary duct; PBM, pancreaticobiliary maljunction; UL, sonographic upper limit of the bile duct by age [10].

^a^ Diameter below cancerous obstruction.

Gallbladder pathologies in patients with PBM and HCPBD with benign gallbladder diseases are listed in Table 3. Diffuse epithelial hyperplasia was found in 67% of patients with PBM and in 33% of those with HCPBD. Precancerous lesions were observed in patients with PBM but not in those with HCPBD.

**TABLE 3:** Gallbladder pathology in patients with PBM or HCPBD with benign gallbladder disease.

Pathology of the gallbladder	PBM	HCPBD
	*n* = 9	*n* = 18
Precancerous lesions		
BilIN 1	1 (11%)	0
Tubular adenoma	1 (11%)	0
Lesions associated with PBM		
Epithelial hyperplasia	6 (67%)	6 (33%)
Adenomyomatosis	1 (11%)	3 (17%)
Metaplasia	0	4 (22%)
Cholesterol polyp	0	2 (11%)
Chronic cholecystitis	8 (89%)	12 (67%)

Abbreviations: BilIN, biliary intraepithelial neoplasia; HCPBD, high confluence of pancreaticobiliary ducts; PBM, pancreaticobiliary maljunction.

The prognoses of the nine patients with PBM and 18 with HCPBD who underwent cholecystectomy for benign gallbladder disease were favorable, without the occurrence of bile duct cancer observed for the median follow‐up periods of 62 (range, 13–91) and 37 (range, 15–117) months, respectively.

## 4 Discussion

The results of this study confirmed a strong correlation between PBM and biliary cancer, particularly gallbladder cancer. Among the known correlations between PBM, congenital biliary dilatation, and biliary cancer, the correlation between congenital biliary dilatation and bile duct cancer was identified first [11]. PBM was identified as an anatomical malformation in patients with congenital biliary dilatation, also called choledochal cysts, and was subsequently recognized to play a central role in the carcinogenesis of the biliary tract through pancreatobiliary reflux [1, 12]. Finally, the discovery of nondilated PBM established a strong association between PBM and an elevated risk of developing gallbladder cancer [13].

A nationwide survey of patients with PBM in Japan showed that 22% of adult patients with congenital biliary dilatation (i.e., dilated PBM) developed biliary cancer in the gallbladder (62%) and dilated bile duct (32%), whereas 42% of adult patients with nondilated PBM developed biliary cancer, primarily in the gallbladder (88%) and rarely in the bile duct (7%) [2]. Our review of the English and Japanese literature revealed that the median prevalence of PBM in patients with gallbladder cancer was 17% (range, 4.6%–50%) (Table 4). The aforementioned frequencies are consistent with the data obtained in our study; however, 33% of the patients with PBM evaluated in this study were undiagnosed (Table 1). This implies that many individuals in the general population with asymptomatic PBM remain undiagnosed, necessitating further research on the true incidence of biliary cancer among patients with PBM.

**TABLE 4:** Reported PBM prevalence among patients with gallbladder cancer.

Author	Country, city	Year	PBM (*n*)	Gallbladder cancer (*n*)	Prevalence (%)	Source
Kato O	Japan, Nagoya	1983	4	8	50.0	Gastrointest Endosc 29:94–8
Kinoshita H	Japan, Osaka	1984	10	48	20.8	Cancer 54:762–9
Kimura K	Japan, Chiba	1985	16	96	16.7	Gastroenterology 89:1258–65
Misra SP	India, New Delhi	1989	2	21	9.5	Gastroenterology 96:907–12
Uchimura M	Japan, Hamamatsu	1991	19	106	17.9	Tan to Sui 12:397–404 (in Japanese)
Suda K	Japan, Yamanashi	1991	18	41	43.9	Tando 5:513–6 (in Japanese)
Chijiiwa K	Japan, Fukuoka	1993	4	37	10.8	Am Surg 59:430–4
Chao TC	Taiwan, Taipei	1995	4	25	16.0	J Clin Gastroenterol 21:306–8
Sandoh N	Japan, Niigata	1997	10	58	17.2	Hepatogastroenterology 44:1580–3
Egami K	Japan, Tama	1998	14	43	32.6	Nihon Ika Daigaku Zasshi 65:7–13
Yoshida T	Japan, Ooita	1999	9	59	15.3	J Am Coll Surg 189:57–62
Elnemr A	Japan, Kanazawa	2001	23	126	18.3	Hepatogastroenterology 48:382–6
Hu B	China, Shanghai	2003	7	54	13.0	Gastrointest Endosc 57:541–5
Kamisawa T	Japan, Tokyo	2006	36	268	13.4	Hepatogastroenterology 53:816–8
Kang CM	Korea, Seoul	2007	10	218	4.6	Can J Gastroenterol 21:383–7
Kim Y	Korea, Ansan	2014	10	197	5.1	Langenbecks Arch Surg 399:1071–6
Park JS	Korea, Seoul	2016	76	1111	6.8	Medicine 95:e3526
Fujimoto T	Japan, Fukuoka	2017	7	22	31.8	J Hepatobiliary Pancreat Sci 24:103–8
Hyvärinen I	Finland, Helsinki	2019	3	18	16.7	Scand J Surg 108:285–90
Kawakami S	Japan, Yamanashi	2021	8	34	23.5	BMC Cancer 21:1245
Present study	Japan, Nagakute	2024	8	49	16.3	
		Median prevalence rate			16.7	
		Mean prevalence rate			19.1	
		Total	298	2639	11.3	

Abbreviation: PBM, pancreaticobiliary maljunction.

While the true prevalence of PBM remains unknown, relevant literature has reported a frequency of 2.2% (range, 0.3%–8.7%) based on ERCP imaging results (Table 5). Yamao et al. [14] reported nine patients with PBM (0.03%) among 27 076 asymptomatic Japanese individuals who underwent sonographic screening for gallbladder wall thickening or bile duct dilation during routine medical checkups. The present study revealed PBM in nine (0.44%) of 2046 patients who underwent cholecystectomy for benign gallbladder disease (Table 1). As gallstones comprised nearly all benign gallbladder diseases and gallstone formation is unrelated to PBM [1], the frequency of 0.44% can be regarded as the prevalence of PBM in the general population.

**TABLE 5:** Reported pancreaticobiliary maljunction (PBM) frequency visualized on endoscopic retrograde cholangiopancreatography (ERCP).

	Country, city	Year	PBM (*n*)	ERCP (*n*)	Frequency (%)	Source
Kato O	Japan, Nagoya	1983	9	300	3.0	Gastrointest Endosc 29:94–8
Kimura K	Japan, Chiba	1985	21	656	3.2	Gastroenterology 89:1258–65
Yamauchi S	Japan, Fukuoka	1987	24	1586	1.5	Am J Gastroenterol 82:20–4
Mori K	Japan, Kanazawa	1993	48	1770	2.7	Hepatogastroenterology 40:56–60
Chijiiwa K	Japan, Fukuoka	1995	29	1325	2.2	Int Surg 80:61–4
Egami K	Japan, Tama	1998	52	1722	3	Nihon Ika Daigaku Zasshi 65:7–13
Wang HP	Taiwan, Taipei	1998	59	680	8.7	Gastrointest Endosc 48:184–9
Kim HJ	Korea, Seoul	2002	30	740	4.1	Gastrointest Endosc 55:889–96
Kamisawa T	Japan, Tokyo	2002	63	2980	2.1	Pancreatology 2:122–8
Hu B	China, Shanghai	2003	10	1067	0.9	Gastrointest Endosc 57:541–5
Nagi B	India, Chandigarh	2003	46	2885	1.6	Abdom Imaging 28:847–852
Kim Y	Korea, Ansan	2014	55	10 255	0.5	Langenbecks Arch Surg 399:1071–6
Park JS	Korea, Seoul	2016	229	46 049	0.5	Medicine 95: e3526
Parlak E	Turkey, Ankara	2022	31	11 589	0.3	Surg Endosc 36:2042–51
		Median frequency			2.2	
		Mean frequency			2.5	
		Total	706	83 604	0.8	

Abbreviations: ERCP, endoscopic retrograde cholangiopancreatography; PBM, pancreaticobiliary maljunction.

A review of the National Clinical Database revealed that cholecystectomies were performed in a median of 135 000 people per year from 2013 to 2018 among 126 million people in Japan [15]. Over the study period of 11 years, 1 485 000 people would have undergone cholecystectomies among 126 million people in Japan. Based on this ratio, the population behind the 2046 patients who underwent cholecystectomy in our department for 11 years was inferred to be 173 600. When a PBM prevalence of 0.44% was applied to 173 600, 764 (0.44%) were likely to have PBM. According to the National Cancer Registry, gallbladder cancer occurred at a median of 6.3 per 100 000 people (0.0063%) per year from 2016 to 2020 in Japan [16]. Based on this incidence rate, gallbladder cancer was estimated to affect 120 out of 173 600 people over the study period of 11 years. When 17% was adopted as the PBM prevalence rate for gallbladder cancers (Table 4) and applied to 120 patients, it was estimated that 20 patients had both PBM and gallbladder cancer. Overall, 20 (2.6%) of 764 patients with PBM had gallbladder cancer in 11 years. Therefore, the incidence of gallbladder cancer among patients with PBM was estimated to be 2.4% per decade, which is one order of magnitude smaller than the rate of 37% reported in a nationwide survey in Japan [2] and 38‐fold higher than the incidence of gallbladder cancer among the general Japanese population (0.063%) over a decade.

Given the high incidence of bile duct cancers among patients with dilated PBM (congenital biliary dilatation), the standard treatment approach involves resection of both the extrahepatic bile duct and the gallbladder. Cholecystectomy is recommended for adult patients with nondilated PBM, but there is no established consensus regarding the necessity of prophylactic bile duct resection [1, 2]. Considering that the incidence of bile duct cancer among patients with nondilated PBM is reported to be one‐tenth that of gallbladder cancer [2], the risk of bile duct cancer was approximately 0.3% for one decade, which may not support the excision of the bile duct in older patients, given that hepaticojejunostomy has a high complication rate. The patients evaluated in this study experienced no adverse events after cholecystectomy alone, as reported previously [3, 17].

This study also demonstrated a probable association between HCPBD and biliary cancer, revealing that 33% of patients with HCPBD in the benign cholecystectomy group exhibited diffuse epithelial hyperplasia, a lesion characteristic of PBM (observed in 67% of patients in this study). Other lesions related to PBM, adenomyomatosis and metaplasia, were also observed in patients with HCPBD (Table 3). Metaplasia was not observed in PBM in this study, but this may be because metaplasia is a local lesion and the entire gallbladder was not examined. Our results support those of Kamisawa et al. [4, 5] who advocated viewing HCPBD as an intermediate variant between normal pancreaticobiliary junctions and PBM. We estimated the prevalence of HCPBD to be approximately 1% of the total population, which is twice that of PBM. In contrast, Kamisawa et al. [4] reported prevalence rates of 1.7% and 2.1% for HCPBD and PBM, respectively, based on an analysis of ERCP images. Occult pancreaticobiliary reflux (OPR), which is characterized by elevated amylase levels in the bile despite a normal pancreaticobiliary junction, is another intermediate variant garnering increasing attention [18]; however, while HCPBD is a morphological intermediate, OPR is a functional intermediate [6]. Diagnosis of OPR requires invasive ERCP; otherwise, it remains undetected until surgery. Visualization of the HCPBD on MRCP is a preoperative precaution to avoid incidental identification and potential rupture of an adenocarcinoma during cholecystectomy, although the diagnosis remains limited.

This retrospective study has some limitations. First, the estimated incidence was based on several assumptions and could not be adjusted for age. A larger cohort of biliary cancers is needed to adjust the risk for age. The presence of adults with silent congenital biliary dilatation was not considered, although its prevalence is likely to be much lower than that of non‐dilated PBM, as most congenital biliary dilatations cause symptoms during childhood and a dilated bile duct is much easier to detect. To elucidate the prevalence of silent dilated PBM, sonographic screening for bile duct dilation during medical checkups is necessary in a cohort much larger than that Yamao et al. did [14]. Second, it is unknown whether the proposed results can be generalized beyond the Asian population, although a report from Finland showed that the prevalence of PBM among Caucasian patients with biliary cancer was the same as that among Asian patients [19]. Third, HCPBD may have been underdiagnosed based on the analysis of MRCP images, which sometimes fail to display the entire length of the NDS when we could not know the exact length of the common channel, even if the patient had a longer common channel; therefore, the increased frequency of HCPBD (3.8%) in patients with bile duct cancer may be attributed to the analysis performed during ERCP in addition to MRCP, which might reflect a frequency closer to real prevalence. It needs a comparative study of MRCP and ERCP on a diagnosis of HCPBD.

In conclusion, this study confirmed the strong association between PBM and gallbladder cancer. PBM was detected in 16% of patients with gallbladder cancer, and gallbladder cancer was found in 44% of patients with PBM. Additionally, PBM was found in seven (0.44%) of the 2046 patients who underwent cholecystectomy for gallstones, which represents the prevalence of PBM in the general population. Combined with the national data on the incidence of cholecystectomy and gallbladder cancer in Japan, the true incidence of gallbladder cancer in patients with PBM has been estimated to be 2.4% over 1 decade, which is 38‐fold higher than that in the general population in Japan. HCPBD, however, was found in approximately 1% of patients with benign gallbladder disease, frequently displaying gallbladder epithelial hyperplasia—such as PBM—and may be associated with biliary cancers.

## Conflicts of Interest

The authors declare no conflicts of interest.

## Data Availability

The data that support the findings of this study are available from the corresponding author upon reasonable request.
